# Transcranial Magnetic Intermittent Theta-Burst Stimulation (iTBS) Enhances Physical Performance in Mixed Martial Arts Athletes: A Pilot Study

**DOI:** 10.3390/brainsci15101047

**Published:** 2025-09-26

**Authors:** Rafael Pereira Azevedo Teixeira, Vanessa Teixeira Müller, Aleksandro Ferreira Gonçalves, Clóvis Albuquerque Maurício, Rodrigo Cunha de Mello Pedreiro, Iordan Emanuel Ferreira Miranda, Victor Vieira, Rodrigo Soares Fortunato, Bianca Miarka

**Affiliations:** 1 Institute of Biophysics Carlos Chagas Filho, Federal University of Rio de Janeiro, Avenida Brigadeiro Trompowski—De 221/222 ao fim Galeão, Rio de Janeiro 21941-590, RJ, Brazil; texera.123@gmail.com (R.P.A.T.); iordan.emanuel@biof.ufrj.br (I.E.F.M.); rodrigof@biof.ufrj.br (R.S.F.); 2 Department of Fights, Laboratory of Psychophysiology and Performance in Sports and Combats, Federal University of Rio de Janeiro, Av. Carlos Chagas Filho, 540—Cidade Universitária, Rio de Janeiro 21941-599, RJ, Brazil; vanessatmuller@yahoo.com.br (V.T.M.); aleksandrofg@gmail.com (A.F.G.); clovisnutesportiva@gmail.com (C.A.M.); rodrigocmp1@gmail.com (R.C.d.M.P.); victorvieira.labppsc@gmail.com (V.V.)

**Keywords:** oxidative stress, transcranial magnetic stimulation, theta burst, mixed martial arts, performance

## Abstract

Background: Transcranial Magnetic Stimulation (TMS) has been employed in athletes from various sports to enhance performance; however, no data have focused on its effects in mixed martial arts (MMA). This study investigated the effects of intermittent theta-burst stimulation (iTBS), an alternative modality of TMS, on motor performance and plasma oxidative-stress biomarkers of ten male mixed martial arts (MMA) athletes. Methods: A randomized controlled trial was conducted with ten professional MMA athletes (aged 18–35 years). Participants were assigned to the experimental (iTBS) or placebo groups. Baseline and post-intervention performance were assessed using the Multiple Frequency Speed of Kick Test (MFSKT) and a Progressive Speed Kick Test (PSKT). Plasma biomarkers of oxidative stress, including thiols, total antioxidant capacity, 8-isoprostane, and carbonylated proteins, were measured before and after the performance tests in both groups. The iTBS was applied to the left primary motor cortex at an 80 motor threshold for the experimental group and at sub-threshold levels for the placebo group. A two-way ANOVA for paired groups, followed by Bonferroni post-hoc tests, were used to analyze the repeated measures, with the significance level set at *p* < 0.05. Results: The findings revealed significant improvements on the MFSKT [25.4 (±1.2) kicks vs. 20.8 (±1.4) kicks] and the PSKT [27.6 (±1.5) vs. 22.4 (±1.7) kicks] in the iTBS group vs. placebo, respectively. No significant differences were observed between the groups in terms of the serum redox balance biomarkers pre- and post-test, suggesting a limited impact on redox homeostasis despite performance enhancement. The placebo group showed no notable changes in either test or biomarker levels. Conclusions: These results highlight the improved physical performance in MMA athletes without altering redox biomarkers in the blood—emphasizing its applicability for neuromodulation in sports.

## 1. Introduction

Transcranial magnetic stimulation (TMS) is a non-invasive technique that uses electromagnetic pulses to modulate neural activity in specific brain regions [1]. Since its introduction by Anthony Barker in 1985, TMS has evolved from single-pulse to repetitive-pulse methods, allowing precise control of cortical excitation or inhibition [2,3]. Among these modalities, theta-burst stimulation (TBS), including intermittent Theta-burst stimulation (iTBS) and continuous theta-burst stimulation (cTBS), has been extensively studied for its distinct effects on cortical excitability and its potential therapeutic applications [4,5]. Intermittent TBS has been used to modulate neuronal activity due to its cortical excitability effect, while cTBS induces cortical inhibition, affecting neurotransmitter levels such as GABA and glutamate [3,6,7].

The applicability and effectiveness of TBS extend beyond psychiatric applications and it is actively being explored in various clinical and non-clinical contexts [8] In psychiatry, TBS has been used to treat mental-health conditions, including treatment-resistant depression [9,10,11] and generalized anxiety disorder [12]. Studies have shown that bilateral TBS can be as effective as unilateral TMS in treating depression and that cTBS may have longer-lasting effects compared to conventional TMS [13,14]. However, the application of TBS is not universally effective across all conditions [15,16]. For instance, in bipolar disorders, TBS has not demonstrated consistent efficacy and may even exacerbate mood instability due to the complex neural dynamics involved in mood regulation [15,16]. These limitations indicate the importance of tailoring TBS protocols to specific physiological or functional goals, ensuring its safety and effectiveness [17,18].

In neurology, TBS has shown promise in enhancing motor recovery in stroke rehabilitation [19], improving motor symptoms in Parkinson’s disease [20], and managing chronic pain and migraines through its ability to modulate cortical excitability [21]. Emerging evidence also indicates the potential of TBS to improve physical and motor performance, particularly through intermittent TBS (iTBS) [19], which facilitates cortical excitability and enhances neuroplasticity. These properties make TBS a tool for optimizing motor functions, both in clinical populations and in healthy individuals seeking to enhance physical performance.

TMS has shown the potential to enhance sports performance [22,23]. By modulating cortical excitability, TMS could reduce fatigue and improve motor function, making it a potential method for optimizing athletic performance [22,23]. In combat sports such as mixed martial arts (MMA), where athletes rely on a combination of strength, endurance, and agility, neuromodulation may provide specific advantages [24,25]. Despite its promising results in other sports, research on the effects of repetitive transcranial magnetic stimulation (rTMS) in MMA athletes remains limited. Investigating how iTBS affects performance and physiological biomarkers in MMA athletes could provide valuable insights and practical applications for enhancing athletic training and performance [26,27]. Thus, this study aimed to evaluate the effects of iTBS on the physical performance and serum redox biomarkers of MMA athletes.

## 2. Materials and Methods

### 2.1. Study Design

**Figure 1:**
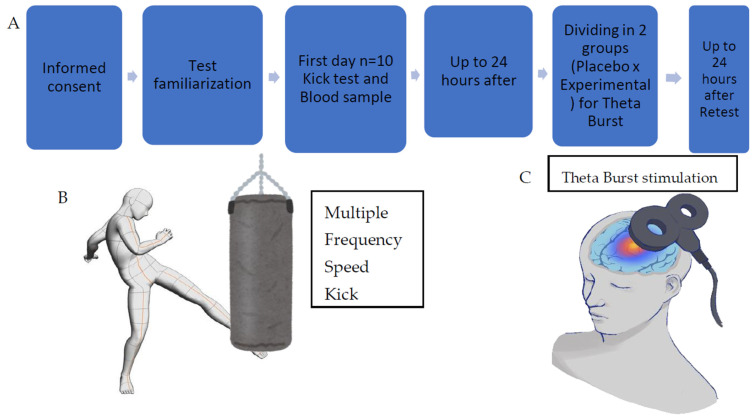
Study design. (**A**) Study fluxogram. (**B**) Design of MFSKT (using low kick adapted to MMA, athletes were instructed not to rotate the support foot; all kicks were recorded via video, and trials in which foot rotation was detected were repeated to ensure standardization; also, the video recordings were used to ensure accurate kick counting). (**C**) Design of theta-burst stimulation. Images created by the author using Canva.com.

This study employed a randomized, double-blind, placebo-controlled design to investigate the effects of iTBS on motor performance and oxidative-stress biomarkers in professional MMA athletes. Following approval from the Ethics Committee of the Clementino Fraga Filho University Hospital (HUCFF, CAAE No. 54465721.4.0000.5257), male MMA athletes, with at least four years of continuous training, were recruited. Athletes were randomly assigned to either the iTBS group or the placebo group using a computerized randomization process. Both the participants and evaluators were blinded to the group allocation. Baseline and post-intervention assessments included the Multiple Frequency Speed of Kick Test (MFSKT) and the Progressive Speed Kick Test (PSKT), performed under standardized conditions. Blood samples were collected pre- and post-tests to analyze oxidative-stress biomarkers. The iTBS intervention targeted the left dorsolateral prefrontal cortex (DLPFC), validated via cranial measurements and Beam systemtools. Both groups received standardized stimulation protocols, with sham stimulation applied to the placebo group. All procedures adhered to strict standardization protocols to ensure consistency and minimize bias. The design of the study followed all of the steps mentioned before (Figure 1).

### 2.2. Participants’ Characteristics and Baseline Measures

Ten male professional mixed martial arts (MMA) athletes, aged between 18 and 35 years, were recruited for this study. All participants had a minimum of four years of continuous training experience and were actively engaged in daily training routines at the same training center, located in Rio de Janeiro, RJ, Brazil. The present study adopted a sample size that is well-aligned with previous research employing neuromodulation techniques in both athletic and clinical populations [19,22]. This sample size reflects established precedents in the field, particularly in studies involving high-performance athletes and complex physiological assessments [19,22]. Given the logistical considerations inherent in collecting neuromodulation and blood biomarker data from elite athletes, the chosen sample represents a realistic and methodologically sound approach for a pilot investigation.

All athletes had been competing in professional MMA bouts for over three years and were engaged in striking-based contact training at least five times per week on a continuous basis. All athletes developed striking skills, including low kicks, through years of regular training, sparring, and competition within the same training environment. They were also familiar with the type of physical performance evaluations used in this study. Despite variations in their original styles, the shared training routines helped to minimize inter-individual variability in motor execution and ensured that the low-kick performance measured was not significantly influenced by differences in their original fighting styles.

Athletes were screened based on the following inclusion criteria: (1) professional MMA fighters with active competition status, (2) no history of neurological or psychiatric disorders, (3) absence of musculoskeletal injuries within the past six months, and (4) no contraindications for transcranial magnetic stimulation (TMS), such as implanted metallic devices or a history of seizures. Participants were excluded if they failed to meet the above criteria or were unable to complete the study protocol. Participants were excluded if they met any of the following exclusion criteria: (1) presence of neurological disorders such as epilepsy, stroke, or traumatic brain injury; (2) diagnosis of psychiatric conditions such as severe depression or bipolar disorder; (3) incompatibility with TMS due to metallic implants, pacemakers, or a history of seizures; (4) recent musculoskeletal injuries or surgeries within the past six months; (5) use of performance-enhancing drugs, recreational drugs, or medications that could influence neuromuscular function or recovery; (6) cardiovascular or metabolic disorders contraindicating physical exertion; (7) acute or chronic illnesses impacting physical performance; and (8) an inability or unwillingness to adhere to the study protocol or provide informed consent.

Eligible participants were randomly allocated into two groups, experimental (iTBS) and placebo, using a computer-generated randomization sequence. The study employed a double-blind design to ensure methodological rigor. The investigator responsible for administering the stimulation procedures received only the athlete’s identification code and had no access to performance data or group assignments. Similarly, the researchers conducting the motor performance assessments (e.g., kick tests) were blinded to the type of stimulation each participant received. This blinding was strictly maintained throughout the study to prevent potential bias during intervention delivery and outcome evaluation. All participants remained in the same waiting area and underwent identical pre- and post-stimulation procedures, regardless of their group assignment. As a result, they were not aware of whether they had received the active or sham stimulation. Therefore, randomization was performed under rigorously controlled double-blind conditions, ensuring that neither the participants nor the evaluators had knowledge of the group allocations at any point during the study. Both groups were matched for key anthropometric characteristics, which are presented in Table 1.

All athletes provided written informed consent before participating in the study, which was approved by the Ethics Committee of the Clementino Fraga Filho University Hospital (HUCFF) under CAAE No. 54465721.4.0000.5257.

### 2.3. Procedures

#### 2.3.1. Familiarization and Standardization

Prior to testing, athletes underwent a familiarization session to ensure that they understood the procedures and could execute them with accuracy and consistency. This phase included detailed verbal instructions, visual demonstrations, and hands-on practice of the performance tests to ensure uniform execution and minimize variability during data collection [28,29]. Athletes were instructed to abstain from caffeine, alcohol, and strenuous physical activity for 24 h prior to each test to standardize the conditions [22]. Compliance with these instructions was verbally confirmed at the start of each session.

#### 2.3.2. Testing Protocol

Prior to data collection, a test was conducted at the laboratory VTM Neurodiagnóstico Institution in Barra da Tijuca, Rio de Janeiro, Brazil, under controlled environmental conditions, with ambient temperature [range: 20–24 °C (68–75 °F)] and humidity (40–60% relative humidity) monitored to ensure consistency in the intervention [22].

#### 2.3.3. Warm-Up

This stage of the study involved both the placebo and experimental groups. Both groups began with a standardized 10 min aerobic warm-up on a stationary bike to elevate the heart rate to 50–60% of their maximum [30], verified using heart rate monitors (Polar H10, Polar Electro, Helsinki, Finland) for accuracy and consistency across participants.

#### 2.3.4. Blood Sampling

Venous blood samples were collected by a licensed phlebotomist immediately after the warm-up (baseline). Samples were drawn from the antecubital vein using a sterile technique, centrifuged at 1500× *g* for 15 min at room temperature, and stored at −80 °C for subsequent biomarker analysis. Sterility and adherence to standardized sampling times minimized variability in oxidative-stress biomarker measurements.

#### 2.3.5. The iTBS Intervention

The iTBS intervention was administered within 24 h after the kick tests and blood collections. It was conducted by a unique neurologist with more than 10 years of experience at a laboratory with controlled conditions utilizing a Neuro-MS/D magnetic stimulator (Neurosoft, Ivanovo, Russia, Anvisa: 80969860026) equipped with an AFEC-02-100-C coil (Neurosoft, Russia).

#### 2.3.6. Left Dorsolateral Prefrontal Cortex (DLPFC) Localization and Preparation

Before the intervention, participants’ skulls were carefully measured using a cap, measuring tape, and permanent markers to accurately estimate the left dorsolateral prefrontal cortex (DLPFC) [23]. To identify the target region corresponding to the DLPFC, first, the researcher used the international 10–20 EEG system, employing F3 as the anatomical reference point. The procedure involved locating the nasion, inion, and left and right tragus landmarks on the participant’s scalp. From these reference points, distances were measured along the sagittal and coronal planes to determine the central point (Cz), after which the F3 location was estimated by identifying the midpoint between F7 and Fz, in accordance with standard 10–20 protocols. This method was selected for its feasibility in applied sport science settings where individual MRI scans are not available [23].

To enhance anatomical estimation and verify the positioning of F3 over the cortical surface, we used Gmsh (bundled with the SimNIBS toolbox) software 2.1 for 3D field visualization. Gmsh was used to visualize the underlying cortical structures and confirm the projection of the stimulation target over the approximate location of the DLPFC. This process involved loading the simulation mesh file (.msh) into Gmsh, selecting the gray matter surface (volume 2, surface 1002), and assessing the field intensity (magnE) on the target area [31]. This targeting was performed following standardized anatomical landmarks to ensure accurate and reproducible coil placement [22,31], ensuring consistency with established neuromodulation protocols [32].

#### 2.3.7. Determination of Motor Threshold

To determine the motor threshold, small magnetic pulses were applied to the cortex, and the resulting contractions of the hand’s thenar muscles were observed. The stimulation intensity was adjusted incrementally until a visible thumb contraction or slight muscle response was achieved. The motor threshold was averaged over multiple trials to ensure reliability [33].

**Figure 2:**
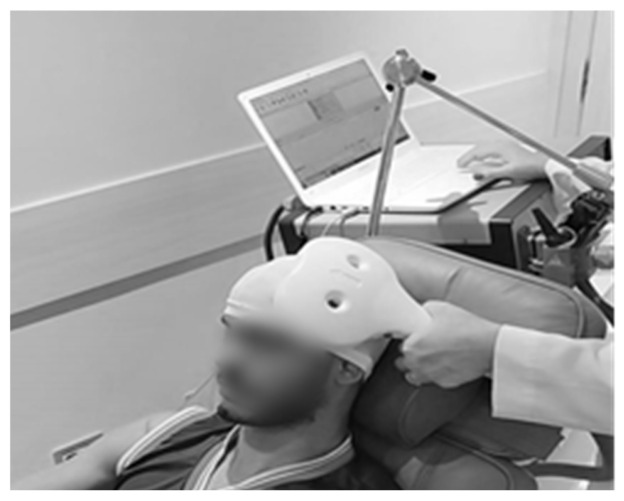
Intermittent TBS or placebo application targeting the DLPFC in an MMA athlete.

Figure 2 shows the precise localization of the DLPFC achieved through cranial measurements and validated motor threshold determination, ensuring consistent and reproducible coil placement during the stimulation protocol.

To ensure consistency, all interventions followed a strict protocol under identical environmental conditions. The intervention duration, coil placement, and stimulation intensity were standardized for both groups, minimizing procedural variability.

#### 2.3.8. Randomization and Group Allocation

Participants were randomized into two groups: the iTBS group and the placebo group. Randomization was carried out using a computerized algorithm to ensure unbiased group allocation. To maintain blinding, athletes were separated into different rooms during the intervention, and those not receiving active stimulation were placed in waiting areas.

#### 2.3.9. Stimulation Parameters

Before the experiment, athletes were separated into different rooms, while those not undergoing the procedure waited in a designated area.

Experimental group: Stimulation was delivered at 80 of the individualized motor threshold using the iTBS protocol. The iTBS consisted of bursts of three pulses at 50 Hz, repeated every 200 ms (5 Hz) for 2 s trains, with 8 s inter-train intervals. The intervention included a total of 600 pulses, lasting approximately 190 s [11,18]. Placebo (sham) group: Sham stimulation was applied at 20% of the motor threshold. The coil was tilted to 90° to minimize effective stimulation while maintaining the sensation of the procedure, ensuring that the placebo condition mimicked the experimental setup [11,18]. Participants were also informed that the study involved different stimulation protocols but were not given information about which protocols were active or placebo. They were unaware of their group assignment throughout the study and still have not reported any suspicion regarding the nature of the stimulation received. Those in the placebo group believed they were undergoing the same procedure as the other participants.

The iTBS and placebo interventions were completed in approximately 3 min for all participants, adhering to the validated parameters established by preceding reports [11,18].

### 2.4. Measures

#### 2.4.1. Multiple Frequency Speed of Kick Test (MFSKT)

For the speed analysis, the MFSKT [28] was adapted specifically for MMA athletes. The original MFSKT involved 5 series of 10 s kicks with 10 s rests between series, which was adapted to 20 s kicks with 10 s rests, simulating the real effort–pause timing of MMA scenarios [34]. Athletes were instructed to use maximum force and speed; otherwise, the test would be invalid. Additionally, they were instructed not to rotate the support foot during kicks in order to standardize the technique. All kicks were video recorded and later reviewed, both to verify the adherence to this instruction and to ensure accurate counting. Trials in which foot rotation was observed were repeated. Performance was determined by the number of kicks per series, with the best series of kicks calculated using: (kick fatigue index (%) = [1 − FSKT1 + FSKT2 + FSKT3 + FSKT4 + FSKT5/best round FSKT × 5] × 100).

#### 2.4.2. Progressive Speed Kick Test (PSKT)

This test followed a progressive kicking protocol [35,36], adapted specifically for MMA athletes. Each stage involved a predefined reduction in time and an increase in the required number of kicks, simulating the effort–pause ratio that occurs in real MMA combat [34].

The test started with athletes performing six kicks within 100 s, with the remaining time allocated for rest. In subsequent stages, four kicks (two for each leg) were added, and the total time decreased [37].

Athletes initiated the test in a fighting stance, beginning with the dominant leg (standardized as the right leg). They alternated legs as required and were instructed to remain in a fighting stance even after finishing the sequence of kicks.

The test ended when (1) the athlete failed to complete the predetermined number of kicks; (2) the athlete failed to perform kicks to the designated area; or (3) the evaluator observed a decrease in force and speed compared to earlier stages, indicating exhaustion. All kicks were recorded and counted to avoid errors.

#### 2.4.3. Blood Collection

Venous blood was collected by a licensed nurse at two time points: baseline, immediately after the warm-up, and post-test, immediately after the MFSKT and PSKT. Blood samples were processed under identical conditions, centrifuged at 1500× *g* for 15 min at room temperature, and stored in 2 mL Eppendorf tubes at −80 °C for subsequent analysis.

#### 2.4.4. Plasma Oxidative-Stress Biomarkers

Reduced thiols: Quantitative determination of reduced thiols in plasma was performed following the protocol by Ellman [38]. This method focuses on the reaction between 5,5′-dithiobis(2-nitrobenzoic acid) (DTNB) and reduced thiols in the sample, forming 2-nitro-5-thiobenzoate (TNB2-), which absorbs at 412 nm. A total of 20 μL of plasma was added to 150 μL of a buffer solution containing 200 mM Tris-HCl and 20 mM EDTA, pH 8.2, with 820 μL of methanol and 10 μL of DTNB (10 mM). The samples were incubated for 15 min at room temperature, then centrifuged at 3000× *g* for 15 min at 25 °C. The supernatant was collected and placed in a 96-well plate for absorbance analysis at 412 nm using a Spectra Max Paradigm 39 plate reader (Molecular Devices, San Jose, CA, USA).

Total antioxidant capacity (TAC): This method is based on the reduction and color change of the 2,2-diphenyl-1-picrylhydrazyl (DPPH) radical. The DPPH radical reduction assay was measured according to preceding protocols [39]. For the assay, 380 μL of sodium phosphate buffer (10 mM, pH 7.4) was mixed with 20 μL of plasma. Then, 400 μL of a 95% ethanol solution containing 0.1 mM DPPH was added. This mixture was incubated for 30 min at room temperature. After incubation, absorbance was measured at 520 nm and compared with a reference consisting of DPPH solution and phosphate buffer only. The absorbance was measured using an Elisa plate reader: Spectra Max Paradigm (Molecular Devices, USA). Results were expressed as the percentage of DPPH oxidation inhibition and in mmol DPPH/mg of protein [40].

The 8-isoprostane levels: They were measured following the manufacturer’s instructions (Cayman Chemical 8-isoprostane ELISA KIT, Cayman Chemicals, MI, USA). The method’s detection limit ranged from 0.8 to 500 pg/mL. Plate readings were taken at 420 nm using a spectrophotometer (Spectra Max Paradigm 39, Molecular Devices, Sunnyvale, CA, USA).

Protein carbonyl content: This was analyzed using the method proposed by authors in the preceding research [41]. Carbonyl groups react with 2,4-dinitrophenylhydrazine (DNPH) to form a stable 2,4-dinitrophenylhydrazone (DNP) conjugated to the protein. A total of 80 μL of plasma was mixed with 80 μL of DNPH (10 mM in 0.5 M H_3_PO_4_) and incubated in the dark for 10 min. Then, 40 μL of NaOH (6 M) was added, followed by further incubation under the same conditions. After incubation, samples were analyzed using an Elisa plate reader: Spectra Max Paradigm (Molecular Devices, USA), at 450 nm. The protein carbonyl concentration was calculated using the molar extinction coefficient of 22,308 mM^−1^ × cm^−1^ for hydrazone derivatives of carbonyl groups and the results were expressed in nmol/mg of protein.

### 2.5. Statistical Analysis

Data were analyzed using SPSS Statistics (version 22.0). A priori power analysis was conducted using G*Power (version 3.1) to ensure an adequate sample size for detecting medium-to-large effect sizes (η^2^p = 0.06) with 80% power and α = 0.05. Before conducting the analyses, data were tested for normality using the Shapiro–Wilk test and for homogeneity of variances using Levene’s test. A two-way repeated measures ANOVA was employed to evaluate the effects of the group (iTBS vs. placebo) and time (pre- vs. post-intervention) on performance metrics and biomarker levels. Partial eta-squared (η^2^p) was calculated to assess the effect sizes. Bonferroni post-hoc tests were conducted for multiple comparisons. All analyses were performed with statistical significance set at *p* < 0.05. Results are reported as the mean ± standard deviation (SD), with graphical representations generated using GraphPad Prism (version 9.0). Also, due to the small sample size and potential violations of normality assumptions, a Wilcoxon signed-rank test (W) was employed for paired within-group comparisons (pre-iTBS vs. post-iTBS), and the Mann–Whitney U test (U) was applied for independent comparisons between groups, especially focusing on delta values (post-iTBS and pre-iTBS to show potential differences). This delta-based approach is particularly useful in studies with small samples, as it focuses on the magnitude of individual changes over time and allows for a more objective comparison between groups; both of these statistical tests were used for the MFSKT and PSKT.

## 3. Results

**Figure 3:**
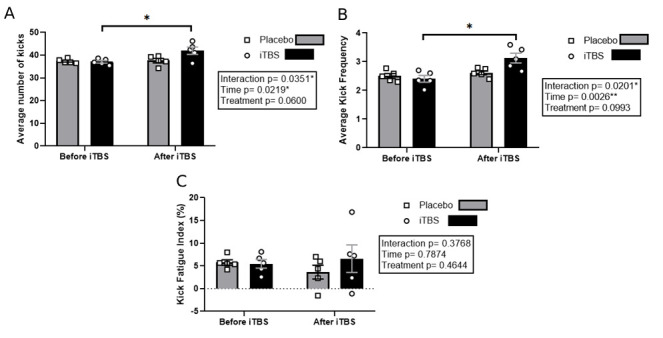
Average kicks of each test after and before theta-burst intervention. (**A**) Differences in the mean number of kicks in the MFSKT pre- and post-intervention with intermittent theta-burst stimulation. (**B**) Mean kick frequency in the Progressive Kick Test for the iTBS and placebo groups. (**C**) Kick Fatigue Index as a percentage of kicks pre- and post-intervention with iTBS. Results are expressed as mean ± standard deviation. * *p <* 0.05. ** *p* < 0.01.

The two-way ANOVA analysis of the MFSKT results revealed significant interactions between the study groups and between the pre- and post-intervention times, but the comparison between groups was not significant (Figure 3A). The iTBS group improved from a mean of 20.8 kicks (95% CI: 19.06–22.54) pre-intervention to 25.4 kicks (95% CI: 23.91–26.89) post-intervention. The placebo group showed stable values from 21.0 (95% CI: 19.39–22.61) to 21.2 kicks (95% CI: 19.46–22.94). The effect size for the intervention (*p* = 0.03, η^2^p = 0.294, large effect) and for time (*p* = 0.02, η^2^p = 0.443, large effect) demonstrated significant differences, and for the treatment, there were no significant differences. In the Progressive Speed Kick Test, significant differences were observed for the intervention (*p* = 0.02, η^2^p = 0.249, large effect) and time (*p* < 0.01, η^2^p = 0.287, large effect), but there were no differences between the study groups, with an increase in the mean kick frequency in the iTBS group after the intervention (Figure 3B). The PSKT showed an increase in the iTBS group from 22.4 kicks (95% CI: 20.29–24.51) to 27.6 kicks (95% CI: 25.74–29.46), while the placebo group did not demonstrate significant difference, with values of 22.6 (95% CI: 20.37–24.83) to 22.9 kicks (95% CI: 20.54–25.26). Additionally, there were no significant changes in the kick fatigue index (Figure 3C).

**Figure 4:**
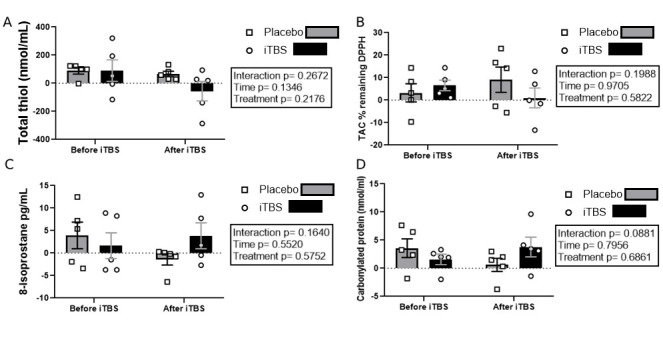
Oxidative-stress biomarkers after and before iTBS intervention. (**A**) Delta of plasma reduced-thiol group concentration in the placebo and iTBS groups before and after the MFSKT and Progressive Kick Test. (**B**) Change in total antioxidant capacity (TAC) in the placebo and iTBS groups, measured before and after the MFSKT and Progressive Kick Test. (**C**) Variation in plasma 8-isoprostane levels in the placebo and iTBS groups, measured before and after the MFSKT and Progressive Kick Test. (**D**) Difference in carbonylated protein levels in the placebo and iTBS groups, assessed before and following the MFSKT and Progressive Kick Test. Results are expressed as mean ± standard deviation.

Regarding plasma oxidative-stress biomarkers, no significant changes were observed for plasma thiol levels (Figure 4A), total antioxidant capacity (Figure 4B), 8-isoprostane levels (Figure 4C), and carbonylated protein (Figure 4D).

The statistical analysis of the iTBS and placebo groups showed no significant within-group increase in the MFSKT scores after the intervention, as assessed by the Wilcoxon signed-rank test (*p* > 0.05). On the other hand, a statistically significant difference was found when comparing the delta values (post- minus pre-intervention) between the iTBS and placebo groups using the statistical Mann–Whitney U test (*p* = 0.0159).

The within-group comparison of the progressive test (pre- vs. post-intervention) in the iTBS group, using the Wilcoxon signed-rank test, did not reach statistical significance (*p* > 0.05), despite the median increasing from 2.35 to 3.09. Furthermore, the placebo group also showed no significant change (with the median variation from 2.49 to 2.59; *p* > 0.05).

Nevertheless, a significant difference was observed when comparing the delta values between the two groups for the progressive test, also using the Mann–Whitney U test (similar to MFSKT), showing a median delta of 0.65 for the iTBS group versus 0.07 for the placebo group (*p* = 0.0079).

## 4. Discussion

The results of the present study revealed that the application of iTBS improved the performance of MMA athletes in the MFSKT compared to the placebo group, with a large size effect. This finding of the observed effects suggests a potentially beneficial impact of iTBS to enhance motor performance, specifically in tasks requiring rapid, repetitive, and precise motor outputs, such as kicking in combat sports. Speed and accuracy are pivotal to success in MMA, making these results particularly relevant to optimizing athletic performance in this discipline [42,43].

The MFSKT is directly related to maximal muscle strength and activation, which are crucial in sports requiring rapid responses and refined motor skills [44]. The observed improvements may stem from iTBS-induced neuroplastic changes, such as enhanced cortical excitability and synaptic efficacy, potentially mediated by long-term potentiation of (LTP)-like effects. The existing literature shows that TMS can enhance muscle power and speed by modulating neuronal activity and improving corticospinal connectivity, reporting both an increase in the maximum voluntary contraction of the leg and a relative gain in muscle strength after 4 weeks of intervention [45]. Our results aligned with the findings of Krogh et al., 2022 [45], who observed increases in the maximum voluntary contraction and muscle strength following TMS interventions. Although our study did not directly measure muscle strength or activation, the enhanced MFSKT performance suggests that iTBS may facilitate neuromuscular efficiency, warranting further investigation with direct neurophysiological measures such as motor-evoked potentials (MEPs).

It is worth noting that although the isolated pre-/post-comparisons within each group did not reach statistical significance, when we focus on the delta comparison between groups, it did reveal significant differences. This supports the potential efficacy of the theta-burst protocol in improving the MFSKT performance. Also, this result reinforces the interpretation that the progressive theta-burst protocol has a potential beneficial impact when it comes to the effect of enhancing performance when compared to the placebo group.

Regarding the PSKT, we observed a higher number of kicks executed by participants who received iTBS. This outcome supports the hypothesis that iTBS can enhance physical endurance and motor performance, even under progressive workload conditions. These results may be partially explained by the DLPFC’s role in cognitive control, motor planning, and fatigue perception, which are critical for sustained high-intensity performance. The DLPFC’s involvement in integrating cognitive and motor processes likely contributed to the observed improvements, highlighting its relevance as a neuromodulation target in combat sports. In previous reports, TMS has demonstrated the ability to enhance motor functions, especially in conditions where these functions are compromised [46]. The observed improvement in performance could also be attributed to iTBS-induced analgesic effects, as previous studies have demonstrated that repetitive magnetic stimulation can reduce pain perception and improve motor function, particularly in compromised conditions [21,47]. The significant differences in PSKT deltas, and the 95% CI shift from 20.29–24.51 to 25.74–29.46 in the iTBS group further strengthen this interpretation. However, further investigations with larger and more diverse samples are warranted to confirm these findings and determine their generalizability across different athletic populations.

Muscle fatigue, resulting from metabolic changes during physical activity, is directly related to strength loss [48]. The progressive taekwondo test conducted in this study aimed to achieve maximal aerobic power, resulting in muscle fatigue and impacting athletes’ performance in terms of the kicks executed [36,49]. In this context, iTBS may not only enhance kick technique and speed but also contribute to a potential reduction in fatigue and pain perception. This hypothesis is supported by previous evidence demonstrating the analgesic properties of TMS, which could have facilitated better performance in the iTBS group [21,47].

Another aspect of the research involved analyzing the effects of iTBS on redox homeostasis biomarkers in MMA athletes, particularly under stress conditions induced by the progressive kick test. The literature indicates that intense exercise can generate oxidative stress [50,51]; the analysis of plasma thiol-group levels did not reveal statistically significant differences between the iTBS and placebo groups. This result contradicts the initial hypothesis that iTBS would enhance antioxidant defense mechanisms under physical stress. One possible explanation is the highly trained state of the athletes, which may have resulted in a robust compensatory antioxidant response independent of the iTBS intervention. Previous research has similarly reported minimal changes in thiol-group levels following resistance training in trained individuals [52], which may suggest a compensatory antioxidant defense among athletes.

Regarding the total antioxidant capacity and lipid peroxidation (8-isoprostane), no significant differences were observed between groups; however, other studies indicate that athletes may experience increases in total antioxidant capacity following intense exercise [53,54,55]. These findings align with those of prior studies suggesting that oxidative-stress responses are highly dependent on exercise intensity and type. Recent combat sport research also found no significant changes in protein carbonyl levels following combat sports simulations, highlighting the complex interplay between exercise and redox homeostasis in Brazilian jiu-jitsu athletes [56].

Despite the absence of biomarker differences, the significant performance improvements and large effect size observed in the iTBS group suggest that neuromodulation effects may not be directly reflected in systemic oxidative-stress markers. Future studies should explore alternative biomarkers, such as inflammatory cytokines or neurotransmitter levels, to gain deeper insights into iTBS-induced physiological changes.

Limitations of this study include the small sample size, which limits the statistical power and generalizability of the findings. Additionally, the absence of direct neurophysiological measures, such as MEPs or electroencephalography (EEG), restricts our ability to confirm the cortical changes induced by iTBS. A lack of long-term follow-up further prevents conclusions regarding the durability of the observed performance benefits. Future research should address these limitations by incorporating larger, more diverse samples and advanced neuroimaging or electrophysiological techniques to validate the mechanisms underlying iTBS effects. Longitudinal studies are also needed to determine whether repeated iTBS sessions can produce cumulative benefits or long-term adaptations. Comparative studies targeting different brain regions (e.g., DLPFC vs. M1) could elucidate the most effective neuromodulatory strategies for optimizing athletic performance.

In practical terms, these findings suggest that iTBS could be integrated into training regimens for combat athletes to enhance performance in tasks requiring speed, precision, and endurance. However, further investigation into the safety, logistical feasibility, and ethical considerations is required before widespread implementation in sports settings.

Currently, this study represents a pioneering effort to investigate the effects of iTBS in the context of MMA, contributing novel insights to the field of neuromodulation and athletic performance. These promising preliminary findings should be interpreted with caution due to the limited sample size, and it is also necessary to focus on ethical considerations when applying neuromodulation techniques such as iTBS in athletic settings. Therefore, there is a need to ensure that when applying iTBS, it does not exacerbate inequalities among athletes by offering an unfair advantage in competitions to those with access to such technology. In addition, it is also important to highlight the improvements that were shown in the MFSKT and Progressive Speed Kick Test performance, highlighting the potential of iTBS as a valuable tool for optimizing combat sport training.

## 5. Conclusions

In conclusion, our findings indicate that iTBS enhanced performance in the MFSKT and PSKT among MMA athletes, indicating its potential applicability as a neuromodulatory tool in combat sports. The present results demonstrate the potential benefits that iTBS has on motor execution, endurance, and speed, all of which are critical components for success in high-intensity athletic contexts. However, no significant changes were observed in oxidative-stress biomarkers following the tests, indicating the complexity of the physiological mechanisms underlying the effects of iTBS and the potential limitations of the biomarkers used in this study. These promising preliminary findings should be interpreted with caution due to the limited sample size, emphasizing the need for further research to verify the relationship between iTBS-induced performance enhancements and physiological adaptations, including the investigation of alternative biomarkers, neurophysiological measures, and different exercise protocols.

## Figures and Tables

**Table 1:** The anthropometric characteristics of 10 professional MMA athletes, divided in two groups, placebo and experimental, expressed as mean ± standard deviation (SD).

	Placebo Group (*n* = 5)	Experimental Group (*n* = 5)
Height (cm)	1.76 ± 0.04	1.75 ± 0.07
Weight (kg)	76.74 ± 4.00	74.56 ± 7.83
BMI (kg·m^−2^)	24.44 ± 1.52	24.17 ± 1.63
Body fat (%)	9.90 ± 1.41	8.26 ± 1.24
Waist circumference (cm)	95.70 ± 3.25	94.80 ± 4.62
Thigh circumference (cm)	54.10 ± 1.32	53.50 ± 3.65

Note: Data were expressed as the mean ± SD. BMI: body mass index.

## Data Availability

The original contributions presented in this study are included in the article. Further inquiries can be directed to the corresponding author.
